# *Zeaxanthin epoxidase 3* Knockout Mutants of the Model Diatom *Phaeodactylum tricornutum* Enable Commercial Production of the Bioactive Carotenoid Diatoxanthin

**DOI:** 10.3390/md22040185

**Published:** 2024-04-19

**Authors:** Cecilie Græsholt, Tore Brembu, Charlotte Volpe, Zdenka Bartosova, Manuel Serif, Per Winge, Marianne Nymark

**Affiliations:** 1 Department of Biology, Norwegian University of Science and Technology, 7491 Trondheim, Norwaytore.brembu@ntnu.no (T.B.); zdenka.bartosova@ntnu.no (Z.B.); manuel.serif@ntnu.no (M.S.); per.winge@ntnu.no (P.W.); 2 Department of Fisheries and New Biomarine Industry, SINTEF Ocean, 7010 Trondheim, Norway; charlotte.volpe@sintef.no

**Keywords:** bioactive carotenoid, diatoxanthin, *Phaeodactylum tricornutum*, CRISPR/Cas9 gene editing, zeaxanthin epoxidase, commercial production line

## Abstract

Carotenoids are pigments that have a range of functions in human health. The carotenoid diatoxanthin is suggested to have antioxidant, anti-inflammatory and chemo-preventive properties. Diatoxanthin is only produced by a few groups of microalgae, where it functions in photoprotection. Its large-scale production in microalgae is currently not feasible. In fact, rapid conversion into the inactive pigment diadinoxanthin is triggered when cells are removed from a high-intensity light source, which is the case during large-scale harvesting of microalgae biomass. Zeaxanthin epoxidase (ZEP) 2 and/or ZEP3 have been suggested to be responsible for the back-conversion of high-light accumulated diatoxanthin to diadinoxanthin in low-light in diatoms. Using CRISPR/Cas9 gene editing technology, we knocked out the *ZEP2* and *ZEP3* genes in the marine diatom *Phaeodactylum tricornutum* to investigate their role in the diadinoxanthin–diatoxanthin cycle and determine if one of the mutant strains could function as a diatoxanthin production line. Light-shift experiments proved that *ZEP3* encodes the enzyme converting diatoxanthin to diadinoxanthin in low light. Loss of ZEP3 caused the high-light-accumulated diatoxanthin to be stable for several hours after the cultures had been returned to low light, suggesting that *zep3* mutant strains could be suitable as commercial production lines of diatoxanthin.

## 1. Introduction

Carotenoids are a diverse group of pigments produced by plants, algae and photosynthetic bacteria that have crucial roles in photosynthesis and protection from photodamage [1]. In humans, carotenoids can have health benefits mainly through their antioxidant effects and are emerging as molecules of vital importance that might offer protection against a variety of chronic diseases like cancer, obesity, cataracts, cardiovascular diseases and neurodegenerative diseases [2,3,4,5]. Additionally, carotenoids like α-carotene and β-carotene are dietary precursors of vitamin A, essential for human eye health and vision. The main applications of these compounds are as dietary supplements, fortified foods, food colours, animal feed, nutraceuticals, pharmaceuticals and cosmetics [6,7,8]. Today, only a few carotenoids are commercially produced, including carotenes (β-carotene and lycopene) and xanthophylls (astaxanthin, canthaxanthin, capsanthin, lutein, zeaxanthin (Zx) and fucoxanthin (Fx)) [9].

Fx is one of the most valuable carotenoids present in the marine environment (market price 40,000–80,000 USD/kg), and it can be extracted from certain groups of marine microalgae or brown seaweed [6,10,11,12,13]. Applications of Fx currently extend from use in the pharma- and nutraceutical industries to animal feed and cosmetic products [6,10]. In algae cells, the main role of Fx is in light harvesting, and low light (LL) conditions increase the production of this carotenoid [10,14,15]. Recent research has highlighted the pronounced bioactivity of another marine carotenoid, diatoxanthin (Dtx), outperforming commercially available carotenoids as potential disease-preventing agents [16,17,18,19]. Antioxidant and anti-inflammatory abilities have been reported, with Dtx found to lower the production of reactive oxygen species (ROS) and pro-inflammatory cytokines in vitro [16,17,18]. Dtx has also been suggested as a potential therapeutic agent in the treatment and/or prevention of the severe inflammatory syndrome related to the SARS-CoV-2 infection [18]. These findings highlight Dtx as a new marine antioxidant and an anti-inflammatory agent of commercial interest.

Dtx is a low-abundance xanthophyll exclusively found in diatoms and a few other groups of microalgae, including dinophytes and haptophytes [20]. Dtx, together with diadinoxanthin (Ddx), comprise the xanthophyll cycle, which is crucial for regulating the flow of energy to photosystem II (PSII) in these algae [20]. The Ddx–Dtx cycle is equivalent to the xanthophyll cycle in higher plants and green algae, where violaxanthin (Vx) is converted to zeaxanthin (Zx) via the intermediate antheraxanthin by violaxanthin de-epoxidase (VDE) [20]. The reverse reaction is catalysed by zeaxanthin epoxidase (ZEP). In diatoms, the qE component (pH- or energy-dependent component) of the photoprotective mechanism non-photochemical quenching (NPQ) of chlorophyll (Chl) *a* fluorescence depends on the high-light (HL)-induced buildup of a transthylakoidal ΔpH, the de-epoxidation of Ddx to Dtx and the presence of specific light-harvesting complex (LHC) proteins of the LHCX class [20,21]. The reverse reaction takes place in low LL. The interconversion between the pigments of the xanthophyll cycle in diatoms is a rapid process, and relaxation of NPQ, including back-conversion of Dtx to Ddx, takes place within minutes after a shift from HL to LL conditions [20,21]. The rapid loss of accumulated Dtx in LL will hamper industrial-scale production of this potentially valuable carotenoid because Dtx will be converted to Ddx during the lengthy harvesting process of the algae biomass. A possible solution is to knock out the gene encoding the enzyme responsible for the epoxidation of Dtx to Ddx, thereby stabilising HL-accumulated Dtx. Transgene-free CRISPR/Cas9 gene editing is possible in the diatom *Phaeodactylum tricornutum*, and this alga is already being produced commercially, making it an obvious choice for studies of genes involved in the Ddx–Dtx cycle [22,23,24]. Enzymes catalysing several of the reactions in the multi-step carotenoid synthesis pathway leading to the formation of Fx, Ddx and Dtx are still unknown, despite great progress in this research area during the last couple of years [25,26,27]. The genome of *P. tricornutum* encodes three proteins belonging to the ZEP family: ZEP1–3 [28,29]. *ZEP1* has recently been found to encode an enzyme essential for the synthesis of Fx [26], whereas *ZEP2* and/or *ZEP3* are candidate genes for encoding the epoxidase converting Dtx to Ddx [26,29,30]. A transmembrane region is predicted to be in the C-terminal domain of ZEP3, which has been hypothesised to be involved in the localisation and/or regulation of the enzyme [20,29]. The roles of ZEP2 and ZEP3 have not yet been confirmed in diatoms, and one or both of these enzymes might also be responsible for an earlier step in the Fx synthesis pathway converting Zx to Vx [26]. Identification of the ZEP responsible for the epoxidation of Dtx to Ddx will be not only of academic interest, filling an important knowledge gap in the carotenoid synthesis pathway of diatoms, but also of commercial interest. A mutant strain where accumulated Dtx remains stable in the cells can facilitate large-scale production of the pigment.

In this context, we created CRISPR/Cas9-mediated knockout mutants of *ZEP2* and *ZEP3* in *P. tricornutum*, aiming to identify the gene encoding the epoxidase responsible for the conversion of Dtx to Ddx. We exposed the mutants to shifts between different light intensities that would trigger the interconversion between the xanthophyll pigments. The carotenoid content, growth, NPQ induction/relaxation and other photosynthetic parameters were compared between *zep* mutants and wild-type (WT) as a response to light treatments. The role of ZEP3 in the Ddx–Dtx cycle was successfully determined, and HL-accumulated Dtx was stabilised.

## 2. Results and Discussion

### 2.1. Phylogenetic and Structural Study of ZEP Genes

**Figure 1:**
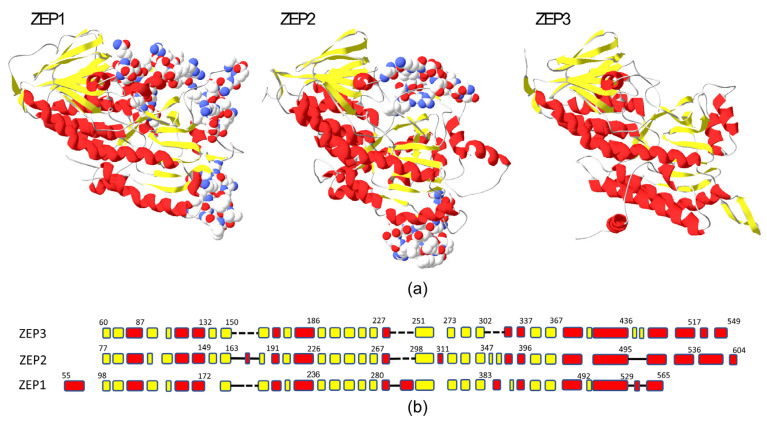
Structural comparison of *P. tricornutum* ZEP proteins. (**a**) The 3D structures of *P. tricornutum* ZEP1, ZEP2 and ZEP3. The protein regions of ZEP1 and ZEP2 that are missing in ZEP3 are shown with side chains in space fill in the ZEP1 and ZEP2 3D models. (**b**) An alignment of the conserved core structure of the ZEP proteins shows the similarities and differences, including the regions missing in ZEP3. The alpha helixes are shown in red, the beta sheets are shown in yellow, and the dashed lines show the missing regions in ZEP3. A solid line indicates loop regions. The numbers indicate amino acid positions at some of the junctions.

**Figure 2:**
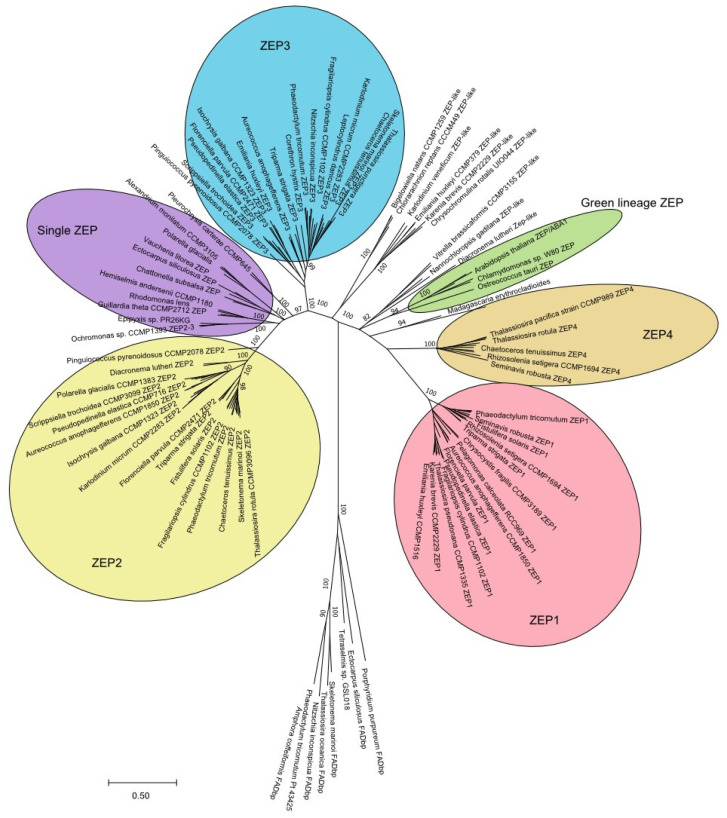
Phylogenetic tree of ZEP proteins. A neighbour-joining (N–J) tree was constructed based on a protein alignment of 158 full-length ZEP proteins. Bootstrap numbers for main clusters with high confidence are shown in the tree. All positions with less than 80% site coverage were eliminated.

The *ZEP* genes are widely distributed in plants and algae, but in terrestrial plants and green algae, they are often represented by a single gene, such as in *Arabidopsis thaliana* (*ZEP/ABA1*) or *Chlamydomonas reinhardtii* (*ZEP1*). In marine algae, the *ZEP* genes have diversified, and several distinct groups have evolved through gene duplications in different phyla [26,31]. In diatoms, most species have three distinct ZEP paralogs: ZEP1, 2 and 3. The *P. tricornutum* gene pairs *ZEP1/VDE-like2* and *ZEP3/VDE* are located next to each other in the genome due to segmental gene duplications. Structurally, the diatom ZEP proteins are similar, but ZEP1 and ZEP2 differ from ZEP3 by having three insert regions that are not found in ZEP3 (Figure 1). In addition, ZEP1 has a conserved N-terminal alpha-helical domain not found in ZEP2 and ZEP3. Some species of Ochrophyta have only one *ZEP* gene, such as the brown algae *Ectocarpus siliculosus* and the raphidophyte *Chattonella subsalsa* (Supplementary File S1). What is common for all single-copy ZEP homologs is that they are structurally more related to ZEP3 proteins. In our search for *ZEP* genes, we also discovered a novel ZEP family in diatoms, named ZEP4 in the phylogenetic tree presented in Figure 2, that may have evolved from a ZEP1-like ancestor. Similar to the ZEP1 proteins, they have a conserved N-terminal domain, which is predicted to form an alpha helix, and they lack the C-terminal alpha-helical part found in ZEP2 and ZEP3. The ZEP4 family has a sparse distribution and is missing in many diatoms, such as *P. tricornutum*, but can be found in several species from the Naviculales, Rhizosoleniales and Thalassiosirales orders. Accession numbers for the ZEP proteins/genes and the distribution of ZEP proteins in various marine phyla are shown in a table in Supplementary File S1.

### 2.2. CRISPR/Cas9-Generated zep2 and zep3 Knockout Mutants

**Figure 3:**
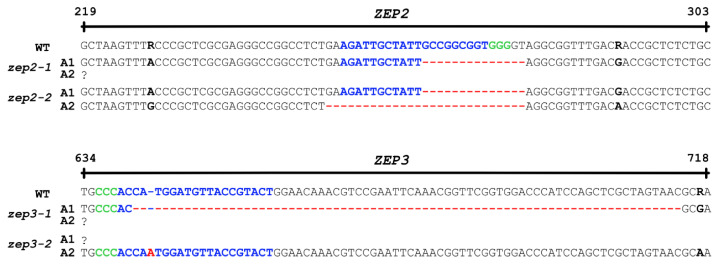
Overview of indels in the two alleles (A1 and A2) of the *ZEP2* and *ZEP3* genes. Blue characters: target sequences; red characters/dashed lines: indels; black characters in bold: polymorphisms; green characters: protospacer adjacent motifs (PAMs). The PAM for *ZEP3* is located on the reverse strand.

Vectors expressing Cas9 and gRNAs targeting either the *ZEP2* or *ZEP3* gene were introduced into *P. tricornutum* cells by bacterial conjugation [22]. Conjugative transformation allows the vector to be maintained as an episome in the diatom cells, avoiding permanent integration of vector DNA into the genome and possible disruption of genetic elements [22,32]. This method also enables the creation of transgene-free mutants because the removal of selection pressure after identification of the mutations of interest causes the cells to lose the CRISPR/Cas9 vector [22]. Mutants containing no foreign DNA might not be legally viewed as GMOs, depending on the individual countries’ gene technology legislation. These mutants are likely to have a higher level of customer acceptance, and the use of the biomass will be subject to fewer restrictions. Most countries outside the EU do not regulate transgene-free gene-edited organisms as GMOs [33], and the EU is currently establishing a new regulatory framework for this type of mutant [34]. Screening of transformants for CRISPR/Cas9-induced mutations resulted in the identification of two *zep2* and two *zep3* knockout lines containing indels of different sizes causing frameshift mutations (Figure 3). Amplification of the two *ZEP* genes by PCR and Sanger sequencing of the PCR products revealed a lack of polymorphism in the sequence for three out of four mutants, indicating that only one allele had been amplified for these mutant lines. This phenomenon is typically caused by large indels or mitotic gene conversion affecting the target site, as observed previously [35,36]. No background signal indicating the presence of a non-mutated WT sequence was observed in any of the mutants where only one allele was amplified by PCR.

### 2.3. Loss of ZEP3 Blocks the Back-Conversion of Diatoxanthin to Diadinoxanthin in Low Light

**Figure 4:**
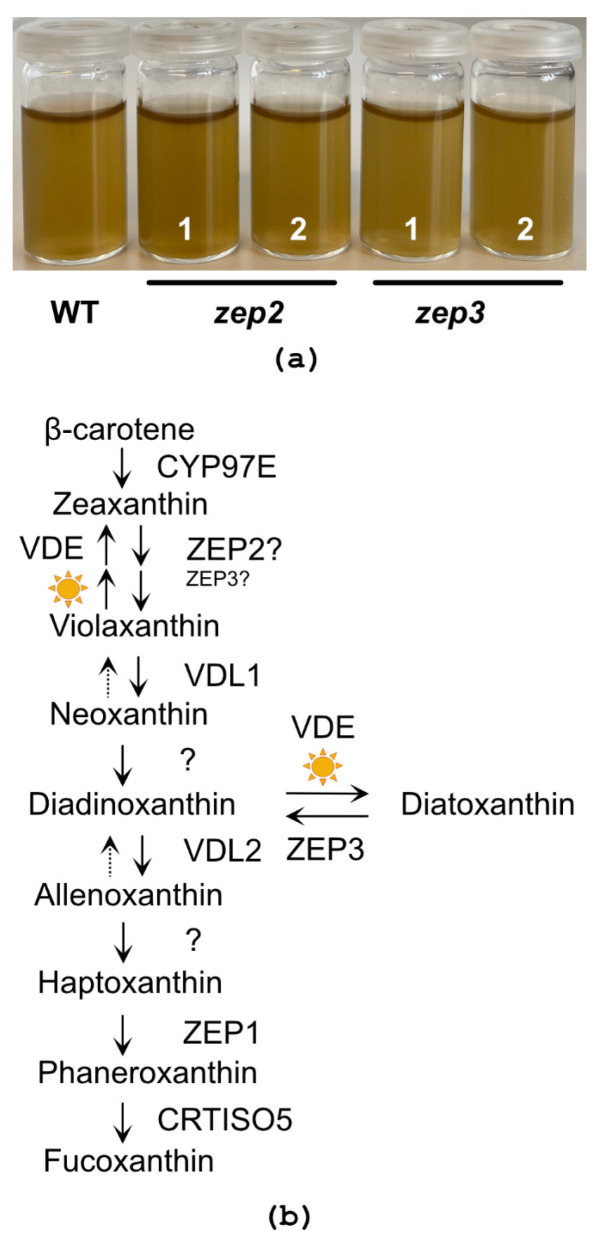
Culture colour and schematic model of the carotenoid biosynthetic pathway. (**a**) Culture colour of low-light (LL)-acclimated WT, *zep2* and *zep3* knockout lines concentrated to 15 million cells, mL^−1^. (**b**) The schematic model of the carotenoid biosynthetic pathway is modified from Bai et al. [26] and Cao et al. [27] and updated with findings from this study described in the main text. The sun symbols indicate reactions taking place under HL conditions. The presence of ZEP3 is necessary for the back-conversion of diatoxanthin (Dtx) to diadinoxanthin (Ddx), and ZEP3 might possibly compensate for a lack of ZEP2 in the zeaxanthin (Zx)–violaxanthin (Vx) reaction step. Abbreviations: CYP97: cytochrome P450 97 family; VDE: violaxanthin de-epoxidase; VDL: violaxanthin de-epoxidase-like; ZEP: zeaxanthin epoxidase; CRTISO5: carotenoid isomerase-like protein 5.

**Figure 5:**
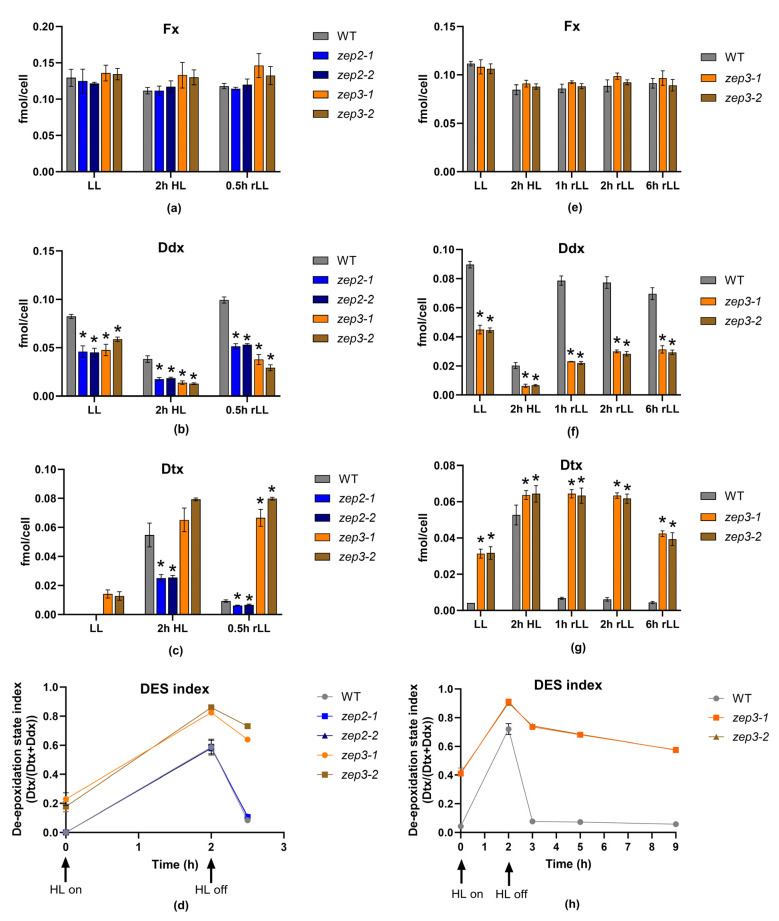
Carotenoid concentration and DES index in WT, *zep2* and *zep3* mutant lines. WT, *zep2* and *zep3* cultures were acclimated to LL and exposed to 2 h of HL before being returned to LL for 0.5 h (rLL). The (**a**) Fx, (**b**) Ddx and (**c**) Dtx cell concentrations are presented as fmol/cell, whereas the (**d**) DES index is calculated as fmol Dtx/fmol (Dtx+Ddx). An additional experiment was performed with only WT and *zep3* lines, where the rLL period was prolonged to 1, 2 and 6 h. The resulting carotenoid concentrations are presented in (**e**) Fx, (**f**) Ddx and (**g**) Dtx. Figure (**h**) describes the DES index pattern resulting from a prolonged recovery time in rLL. All results are presented as the means of three biological replicates ± SD. Asterisks describe significant differences between carotenoid concentrations in *zep* mutants and WT, as indicated by a two-way ANOVA with Dunnett’s multiple comparison tests (*p* < 0.05).

Fx is responsible for the golden-brown colour of diatoms. Green phenotypes have been reported for diatom mutants of the carotenoid synthesis pathway being completely devoid of Fx, and for mutants with strongly reduced levels of Fx as a result of lowered levels of pigment-binding proteins [26,27,37,38]. An initial examination of the *zep2* and *zep3* mutant cultures did not reveal a colour change from brown to green in either mutant (Figure 4a), indicating that Fx synthesis was not affected and that neither ZEP2 nor ZEP3 are essential for the conversion of Zx to Vx (Figure 4b). To determine the effect of knocking out the *ZEP2* and *ZEP3* genes on the carotenoid synthesis in *P. tricornutum*, LL-acclimated WT and mutant cells were exposed to HL for 2 h before being returned to LL (rLL) for 0.5 h. Material harvested at the different time points was subjected to high-performance liquid chromatography (HPLC) analyses to determine the pigment content. The analyses showed that the Fx and Chl concentrations were at WT levels in both mutants, confirming the visual impression of the mutant lines (Figure 5a and Figure S1). In contrast, significant and mutant-specific differences were identified for Ddx and Dtx when compared to WT (Figure 5b,c). The Ddx and Dtx concentrations in *zep2* mutants were consistently lower than WT at all light treatments, resulting in a smaller pool of photoprotective pigments. However, the *zep2* mutants displayed a close to identical de-epoxidation state (DES) index (Dtx/(Dtx+Ddx)) pattern as WT as a response to the shifts in light intensities (Figure 5d), indicating that the cyclic interconversion between Ddx and Dtx is unaffected by the loss of ZEP2. Despite the *zep2* mutants displaying WT levels of Fx and a functional Ddx–Dtx cycle, the reduced pool of Dtx+Ddx still implies that ZEP2 plays a role in the synthesis of carotenoids in diatoms. The mild *zep2* phenotype might be explained by ZEP3 being able to compensate for the loss of ZEP2 by catalysing the Zx to Vx reaction, although with a lower efficiency than ZEP2.

In contrast to the *zep2* strains, the *zep3* strains show higher Dtx and higher DES index levels than WT throughout the experiments and, most strikingly, an inability to back-convert Dtx accumulated during the HL treatment to Ddx when returned to LL conditions (Figure 5b–d). In *zep3* mutants, Dtx is already abundant in LL conditions. In green algae, where Zx and Vx comprise the xanthophyll cycle, a similar phenotype has been reported for *zep* knockout mutants, where Zx accumulates in all light conditions [39,40]. To further investigate the stability of accumulated Dtx in the *zep3* strains, an additional experiment was performed, where material was harvested 1, 2 and 6 h after the cultures had been returned to LL (Figure 5e–h). These results corroborated our initial findings that ZEP3 is the enzyme responsible for the epoxidation of Dtx to Ddx (Figure 5f–h). The Dtx concentration in the *zep3* strains remained at HL levels after 1 and 2 h of rLL (Figure 5g) and was ten times higher than in the WT at the same time points. The maximum Dtx concentrations in the *zep3* strains in our study were also five times higher than the concentrations reported using transgenic *P. tricornutum* double (*Vde/Vde-related* (*Vdr*)) and triple (*Vde*/*Vdr*/*Zep3*) overexpression lines (light intensity: 90 µmol photos m^−2^ s^−1^) [41]. An average decline in Dtx levels of approximately 40% could, however, be observed at the 6 h rLL time point compared to HL levels (Figure 5g), but the decline coincided with a similar increase in cell number, suggesting that Dtx had been distributed between daughter cells. The above-described results suggest that the yield of Dtx produced from a *zep3* mutant culture will stay high during harvesting of the biomass in an industrial setting.

### 2.4. Loss of ZEP3 Inhibits Relaxation of the Photoprotective Mechanism NPQ

**Figure 6:**
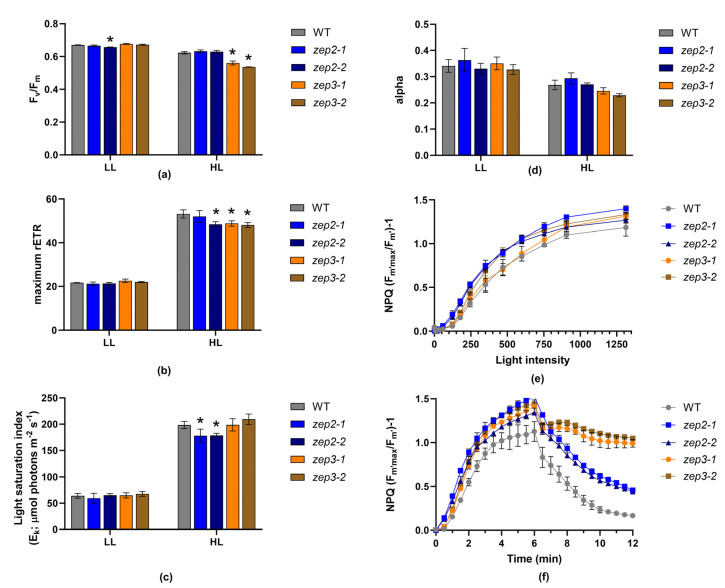
Photophysiological responses of WT, *zep2* and *zep3* mutants. (**a**) The photosynthetic (PSII) efficiency (F_v_/F_m_), (**b**) the photosynthetic capacity (rETR_max_), (**c**) the light saturation index (E_k_) and (**d**) the maximum light utilisation coefficient (alpha) in cells acclimated to either LL or HL. Asterisks describe significant differences between *zep* mutants and WT, as indicated by a two-way ANOVA with Dunnett’s multiple comparison tests (*p* < 0.05). NPQ was calculated both as (**e**) a function of increasing blue light intensity (0–1313 µmol photons m^−2^ s^−1^) and as (**f**) a function of time, where the cells were exposed to 6 min of high-intensity blue light (470 µmol photons m^−2^ s^−1^), immediately followed by a 6 min recovery period in low-intensity blue light (8 µmol photons m^−2^ s^−1^). All results are presented as the means of three biological replicates ± SD.

To investigate the effect of loss of ZEP2 and ZEP3 at the physiological level, the growth rate and photosynthetic performance of *zep2* and *zep3* mutants were compared to WT using cultures acclimated to LL or HL. The photophysiological effects were assessed using Chl *a* variable fluorescence for calculations of the photosynthetic (PSII) efficiency (maximum quantum yield, F_v_/F_m_), the photosynthetic capacity (maximum relative electron transport rate, rETR_max_), the maximum light utilisation coefficient (the slope of the photosynthesis versus irradiance curves, alpha) and the light saturation index (E_k_ = rETR_max_/alpha) at the two different light conditions (Figure 6a–d). E_k_, alpha and rETR_max_ were derived from rapid light curves. These measurements revealed no or minor differences in photosynthetic performance between *zep2* and *zep3* mutants and WT. The similar photosynthetic performance in all strains is in line with the highly similar cell division rates of mutants and WT in LL and HL (Table 1). Because of the possibility of the *zep3* mutant strains being of interest for commercial cultivation, we also investigated the growth of light fluctuating on a millisecond scale, simulating conditions experienced in a photobioreactor (PBR; Table 1). PBR light conditions did not induce statistically significant growth differences under these conditions either, supporting the possibility of industrial cultivation of *zep3* strains. Also, no statistical differences were found between the maximum NPQ values in *zep* mutants compared to WT—neither when NPQ was induced by a stepwise increase in blue light intensity, nor when NPQ was triggered by exposure to constant high-intensity blue light (Figure 6e,f). In contrast, the relaxation behaviour of NPQ in low-intensity blue light was mutant-specific and clearly deviated from WT (Figure 6f). A lack of ZEP3 strongly inhibited the relaxation of NPQ. After a small but rapid decline within the first minutes in very dim light, the NPQ relaxation curve of *zep3* flattened out, and the NPQ level remained at approximately 70% of the maximum level at the end of the relaxation period. The inhibition of NPQ relaxation correlates with the *zep3* mutants’ inability to back-convert Ddx to Dtx when returned to LL and the importance of the presence of Dtx for the performance of NPQ in diatoms [20,21]. Still, the modest relaxation of NPQ despite the stable content of Dtx indicates the additional presence of a short-lived fluorescence quenching mechanism independent of a decline in Dtx [42]. This fast (<1 min) relaxation component has previously been described in the centric diatom *Cyclotella meneghiniana*, and more recently, it has also been observed in *P. tricornutum* (pennate diatom), where it was believed to be absent [42,43,44,45]. This fast NPQ mechanism seems to be dependent on the concentration of Dtx and has been interpreted as the relaxation of part of the steady-state Dtx-dependent quenching [42]. However, further studies are needed to clarify the mechanisms behind this and if differences are present between centric and pennate diatoms. The NPQ relaxation pattern of *zep2* was more similar to that of WT but less efficient. NPQ in *zep2* relaxed to approximately 30% of maximum levels, whereas the equivalent number for WT was 15%. The DES index of the *zep2* mutants was close to identical to that of WT and did not correlate with the slower NPQ relaxation behaviour of *zep2*.

## 3. Materials and Methods

### 3.1. Structural Comparison and Phylogenetic Analyses of ZEP Proteins

The pdb files of the predicted 3D structures of *P. tricornutum* ZEP1, ZEP2 and ZEP3 were downloaded from the AlphaFold2 server (https://alphafold.ebi.ac.uk/, accessed on 19 February 2024) [46,47]. The Swiss PDB Viewer 4.1 (https://spdbv.unil.ch/) was used to view and analyse the proteins [48]. In the 3D models, the non-conserved regions, including the leader peptides with chloroplast-targeting motifs and transit peptides, were excluded from the models. For the phylogenetic analyses, a protein alignment of 158 full-length ZEP proteins was produced using ClustalW [49] and manually refined using GeneDoc software version 2.7.000 (http://nrbsc.org/gfx/genedoc, accessed on 19 February 2024). The phylogenetic analysis was conducted in MEGA11 software (Version 11, https://www.megasoftware.net/, accessed on 19 February 2024) [50] using the maximum likelihood method and the Le_Gascuel_2008 model [51]. An unrooted radial maximum-likelihood tree was produced, where a group of flavin-binding proteins distantly related to the ZEP proteins served as an outgroup. The percentage of trees in which the associated taxa clustered together is shown by numbers; in total, 100 bootstrap replicates were made. Bootstrap numbers for main clusters with high confidence are shown in the tree. The rate variation model allowed for some sites to be evolutionarily invariable ([+I], 2.15% sites). The tree is drawn to scale, with branch lengths measured in the number of substitutions per site. All positions with less than 80% site coverage were eliminated. There were a total of 436 positions in the final dataset.

### 3.2. CRISPR/Cas9 Gene Editing of the ZEP2 and ZEP3 Genes

Knockout mutations in the *ZEP2* (Phatr2_5928) and *ZEP3* (Phatr2_10970) genes in *P. tricornutum* were generated using the CRISPR/Cas9 tool adapted for gene editing in diatoms [22,52,53]. The pPtPuc3m diaCas9_sgRNA vector expressing Cas9 and single-guide RNAs (sgRNAs) targeting *ZEP2* or *ZEP3* was delivered to *P. tricornutum* cells by bacterial conjugation, as described by Sharma and coworkers [22]. The cloning of gene-specific adapters into the sgRNA of the pPtPuc3m diaCas9_sgRNA vector was performed as described in the published protocol by Nymark et al. [53]. The *P. tricornutum* cells that were subjected to CRISPR/Cas9 gene editing were derived from clone Pt1 8.6 (CCMP632) from the culture collection of the Provasoli–Guillard National Center for Culture of Marine Phytoplankton, Bigelow Laboratory for Ocean Sciences, East Boothbay, Maine, USA. Screening, identification and isolation of cells containing bi-allelic mutations in *ZEP2* and *ZEP3* were performed as described previously [52,53]. *ZEP2-* and *ZEP3*-specific oligonucleotides used for the creation of the adapters inserted into the sgRNA of the pPtPuc3m diaCas9_sgRNA vector and primers used for screening purposes are presented in Table 2.

### 3.3. Light Conditions

*P. tricornutum* WT, *zep2* (*zep2-1*, *zep2-1*) and *zep3* (*zep3-1*, *zep3-2*) mutant strains were grown at 15 °C in f/2 medium [54] made with 0.2 µm sterile filtered and autoclaved seawater from the Trondheim fjord. Experimental light conditions were either continuous white light at 35–40 μmol photons m^−2^ s^−1^ (low light (LL)) or high light (HL) at 450–500 μmol photons m^−2^ s^−1^. Cultures for estimation of cell division rates and measurements of photosynthetic parameters were cultivated at LL or HL in a growth chamber equipped with neutral white LEDs (4000 K). Because of the potential industrial relevance of the mutant strains, their growth rate was also investigated in light conditions mimicking the light perception of one cell in a PBR. Nanocosm, a LED-based miniature PBR system [55], was programmed to mimic PBR conditions by fluctuating from darkness to 200 µmol photons m^−2^ s^−1^ in a time scale of milliseconds based on the findings of Luo and coworkers [56]. The light intensity was measured with a ULM-500 (Walz, Effeltrich, Germany) light meter equipped with a spherical sensor. Cultivation of algae cells for pigment analyses was performed in a growth room where LL was provided by fluorescent cool daylight tubes (colour code 865/6500 K), whereas a full-spectrum LED lamp (5500 K) was used to achieve HL conditions. The light intensity in the growth room was measured using a LI-250A light meter (LI-COR Biosciences, Lincoln, NE, USA).

### 3.4. Growth Rates

Cell division rates were estimated in WT, *zep2* and *zep3* mutant lines (three biological replicates for each line) acclimated to either LL, HL or PBR conditions. The cells were grown in 24-well plates (2 mL of culture in each well) at a starting concentration of 30,000 cells mL^−1^. Growth was measured indirectly by recording the daily increase in in vivo Chl *a* fluorescence (IVF; Ex: 460 nm, Em: 680 nm) for nine days. IVF was measured using a Tecan Spark plate reader at five different points in each well. The averaged IVF values were used to plot growth curves, and the cell division rates were calculated from the exponential part of the curves.

### 3.5. Measurements of Photosynthetic Parameters

The photosynthetic parameters F_v_ (F_m_ − F_0_)/F_m_ (photosynthetic efficiency), the maximum relative electron transport rate (rETR_max_; photosynthetic capacity), the maximum light utilisation coefficient (alpha) and the light saturation index (E_k_ = rETR_max_/alpha) were calculated based on measurements of variable in vivo Chl *a* fluorescence using a Multi-Color-PAM fluorometer (Walz, Germany). The instrument was equipped with a Peltier cell (US-T/S, Walz) to keep the temperature constant at 15 °C (±0.2 °C) during measurements. Rapid light curves (RLCs) were obtained by exposing the samples to 14 stepwise increasing irradiances of 0–1313 µmol photons m^−2^ s^−1^ (blue (440 nm) measuring and actinic light) after a 5 min incubation period in darkness. Each light step in the RLCs lasted 30 s. The cardinal points (alpha, ETR_max_ and E_k_) of the light curves were determined by the build-in fitting routine of the PamWin-3 software package (ver. 3.20). NPQ was calculated as a function of the stepwise increasing light intensity from F_m_ and F_m′_ values generated during measurements of the rapid light curves as (F_m_/F_m′_) − 1. Additionally, NPQ induction and relaxation were investigated by exposure of LL-acclimated cells to 470 µmol photons m^−2^ s^−1^ of blue light for 6 min, immediately followed by a 6 min incubation period at 8 µmol photons m^−2^ s^−1^ of blue light. The initial F_m_ value was measured after 5 min of dark incubation, and F_m′_ was measured every 30 s during the light treatments. Cells for measurements of photosynthetic parameters were grown in sterile cell culture flasks with a volume of 30 mL.

### 3.6. Diatoxanthin in Vivo Stability Experiment (Pigment Analyses)

LL-acclimated WT, *zep2* and *zep3* mutant strains were exposed to 2 h of HL before being returned to LL (rLL) conditions for 0.5 h. The experiment was repeated with WT and *zep3* mutants, where the rLL treatment was prolonged to 1, 2 and 6 h. Three biological replicates were included for each line in both experiments. For each biological replicate, samples for pigment analyses were taken successively from the same culture. Cell concentrations at the time of harvesting were 0.6–1.5 × 10^6^ cells mL^−1^. Cell numbers were determined by flow cytometry using a NovoCyteTM flow cytometer (Agilent, Santa Clara, CA, USA) as described previously [52] or a Multisizer 4e Coulter Counter (Beckmann Coulter, Indianapolis, IN, USA). Pigment analyses were performed by HPLC using a Hewlett-Packard HPLC 1100 Series system (Agilent, Santa Clara, CA, USA) as described previously [38,57]. Cells for pigment analyses were grown in sterile cell culture flasks in a volume of 100 mL.

### 3.7. Statistics

A two-way ANOVA with Dunnett’s multiple comparison tests was carried out using GraphPad Prism software (version 10.1.0, GraphPad, Boston, MA, USA) to determine if there were significant differences (*p* < 0.05) between the pigment concentrations and NPQ values in *zep2* and *zep3* mutants compared to the WT. A one-way ANOVA with Dunnett’s multiple comparison tests was used to determine if there were significant differences (*p* < 0.05) between the growth rates in *zep2* and *zep3* mutants compared to the WT using the same software.

## 4. Conclusions

In this study, we aimed to genetically engineer the marine diatom *P. tricornutum* to enable industrial production of the bioactive pigment diatoxanthin. We used the CRISPR/Cas9 technology for targeted disruption of the *ZEP2* and *ZEP3* genes. ZEP2 and/or ZEP3 had previously been suggested to be responsible for the enzymatic conversion of Dtx to Ddx in LL conditions. Based on the combined results, we conclude that the presence of ZEP3 is essential for the conversion of Dtx to Ddx in *P. tricornutum*. ZEP2 is unable to compensate for a lack of ZEP3, meaning that ZEP2 and ZEP3 do not have overlapping functions in the Ddx–Dtx cycle. The role of ZEP2 in the carotenoid synthetic pathway of diatoms was not revealed by our study, and a *zep2–zep3* double knockout mutant needs to be investigated to determine its function in catalysing the transformation of Zx to Vx. The stability of HL-accumulated Dtx for hours after the removal of *zep3* mutants from the HL source and the WT-like photosynthetic performance and growth rates at all tested light conditions indicate that such strains might function as a commercial-scale production line for the bioactive carotenoid Dtx from diatoms. The feasibility of large-scale cultivation and production of Dtx using the *zep3* strains created in this study will be further investigated together with microalgae companies in an ongoing research project funded by the Research Council of Norway.

## Figures and Tables

**Table 1:** Growth rates of WT, *zep2* and *zep3* mutant strains acclimated to LL, HL or PBR conditions. The maximum cell divisions per day during the exponential phase were calculated from three biological replicates of WT, *zep2* and *zep3* mutant lines acclimated to LL (35–40 µmol photons m^−2^ s^−1^), HL (450–500 µmol photons m^−2^ s^−1^) or rapidly fluctuating light simulating photobioreactor light conditions (PBR). Values are presented as the mean ± SD. The growth rates in mutant strains did not show statistically significant differences compared to WT in any light condition (one-way ANOVA with Dunnett’s multiple comparison tests).

	LL	HL	PBR
WT	1.21 ± 0.17	1.80 ± 0.30	1.65 ± 0.28
*zep2-1*	1.25 ± 0.14	1.71 ± 0.26	1.34 ± 0.16
*zep2-2*	1.29 ± 0.12	1.86 ± 0.12	1.52 ± 0.22
*zep3-1*	1.25 ± 0.06	1.76 ± 0.37	1.45 ± 0.14
*zep3-2*	1.23 ± 0.05	1.78 ± 0.31	1.28 ± 0.08

**Table 2:** Adapter sequences, PCR, high-resolution melting (HRM) analyses and sequencing primers.

Oligo or Primer Name	Orientation	Sequence (5’→3’)	Purpose
ZEP2-PAM2_F	Forward	TCGAGCGCGTGGAGATACGGAGAG	Adapter for sgRNA
ZEP2-PAM2_R	Reverse	AAACCTCTCCGTATCTCCACGCGC	
ZEP3-PAM2_F	Forward	TCGAAGTACGGTAACATCCATGGT	Adapter for sgRNA
ZEP3-PAM2_R	Reverse	AAACACCATGGATGTTACCGTACT	
ZEP2-PAM12_scrF	Forward	GAATCGATCTGAATTGGCTACG	Screening for *zep2* (508 bp amplicon)
ZEP2-PAM12_scrR	Reverse	CGGTGAAAGTGAACTTGTCCAT	
ZEP3-PAM2_scrF	Forward	GCACCACCTTCGAGCAATGT	Screening for *zep3* (643 bp amplicon)
ZEP3-PAM2_scrR	Reverse	TCGCCAGCGAAAACCGTGTA	
ZEP2-PAM2_hrmF	Forward	CTCCGGAAGACGTTGCCTTTGA	HRM for *zep2* (146 bp amplicon)
ZEP2-PAM2_hrmR	Reverse	TCTCGTACACCGTCACGTCGAA	
ZEP3-PAM2_hrmF	Forward	TGGTCTTTCCTTGGCCAAGGTT	HRM for *zep3* (111 bp amplicon)
ZEP3-PAM2_hrmR	Reverse	GTTACTAGCGAGCTGGATGGGT	
M13-rev (−29)	Reverse	CAGGAAACAGCTATGAC	Sequencing primer

## Data Availability

The ZEP2 and ZEP3 genes have Draft ID Phatr2_5928 and Phatr2_10970, respectively. AlphaFold2 accession numbers are ZEP1: B7FYW4, ZEP2: B7FQV6 and ZEP3: B7FUR7. *Zep2* and *zep3* mutant strains can be shared for research purposes. Raw data generated in the present study used for the calculation of pigment concentrations, photophysiological parameters and cell division rates are available on request.

## Supplementary Materials

The following supporting information can be downloaded at https://www.mdpi.com/article/10.3390/md22040185/s1, Figure S1: Chlorophyll (Chl) *a* and Chl *c*_1_ + *c*_2_ concentration in WT, *zep2* and *zep3* mutant lines; Supplementary File S1: Distribution of ZEP proteins in various marine phyla.

## References

[1] Sandmann G. (2021). Diversity and Origin of Carotenoid Biosynthesis: Its History of Coevolution towards Plant Photosynthesis. New Phytol..

[2] Meléndez-Martínez A.J. (2019). An Overview of Carotenoids, Apocarotenoids, and Vitamin A in Agro-Food, Nutrition, Health, and Disease. Mol. Nutr. Food Res..

[3] Ren Y., Sun H., Deng J., Huang J., Chen F. (2021). Carotenoid Production from Microalgae: Biosynthesis, Salinity Responses and Novel Biotechnologies. Mar. Drugs.

[4] Kabir M.T., Rahman M.H., Shah M., Jamiruddin M.R., Basak D., Al-Harrasi A., Bhatia S., Ashraf G.M., Najda A., El-kott A.F. (2022). Therapeutic Promise of Carotenoids as Antioxidants and Anti-Inflammatory Agents in Neurodegenerative Disorders. Biomed. Pharmacother..

[5] Seth K., Kumar A., Rastogi R.P., Meena M., Vinayak V. (2021). Harish Bioprospecting of Fucoxanthin from Diatoms—Challenges and Perspectives. Algal Res..

[6] Pocha C.K.R., Chia W.Y., Chew K.W., Munawaroh H.S.H., Show P.L. (2022). Current Advances in Recovery and Biorefinery of Fucoxanthin from *Phaeodactylum Tricornutum*. Algal Res..

[7] Sathasivam R., Ki J.-S. (2018). A Review of the Biological Activities of Microalgal Carotenoids and Their Potential Use in Healthcare and Cosmetic Industries. Mar. Drugs.

[8] Singh T., Pandey V.K., Dash K.K., Zanwar S., Singh R. (2023). Natural Bio-Colorant and Pigments: Sources and Applications in Food Processing. J. Agric. Res..

[9] Ávila-Román J., García-Gil S., Rodríguez-Luna A., Motilva V., Talero E. (2021). Anti-Inflammatory and Anticancer Effects of Microalgal Carotenoids. Mar. Drugs.

[10] Peng J., Yuan J.-P., Wu C.-F., Wang J.-H. (2011). Fucoxanthin, a Marine Carotenoid Present in Brown Seaweeds and Diatoms: Metabolism and Bioactivities Relevant to Human Health. Mar. Drugs.

[11] Galasso C., Corinaldesi C., Sansone C. (2017). Carotenoids from Marine Organisms: Biological Functions and Industrial Applications. Antioxidants.

[12] Bae M., Kim M.-B., Park Y.-K., Lee J.-Y. (2020). Health Benefits of Fucoxanthin in the Prevention of Chronic Diseases. Biochim. Biophys. Acta Mol. Cell Biol. Lipids.

[13] Leong Y.K., Chen C.-Y., Varjani S., Chang J.-S. (2022). Producing Fucoxanthin from Algae—Recent Advances in Cultivation Strategies and Downstream Processing. Bioresour. Technol..

[14] Nymark M., Valle K.C., Brembu T., Hancke K., Winge P., Andresen K., Johnsen G., Bones A.M. (2009). An Integrated Analysis of Molecular Acclimation to High Light in the Marine Diatom *Phaeodactylum Tricornutum*. PLoS ONE.

[15] Brown J.S. (1988). Photosynthetic Pigment Organization in Diatoms (Bacillariophyceae). J. Phycol..

[16] Konishi I., Hosokawa M., Sashima T., Maoka T., Miyashita K. (2008). Suppressive Effects of Alloxanthin and Diatoxanthin from *Halocynthia Roretzi* on LPS-Induced Expression of pro-Inflammatory Genes in RAW264.7 Cells. J. Oleo Sci..

[17] Pistelli L., Sansone C., Smerilli A., Festa M., Noonan D.M., Albini A., Brunet C. (2021). MMP-9 and IL-1β as Targets for Diatoxanthin and Related Microalgal Pigments: Potential Chemopreventive and Photoprotective Agents. Mar. Drugs.

[18] Sansone C., Pistelli L., Del Mondo A., Calabrone L., Fontana A., Noonan D.M., Albini A., Brunet C. (2022). The Microalgal Diatoxanthin Inflects the Cytokine Storm in SARS-CoV-2 Stimulated ACE2 Overexpressing Lung Cells. Antioxidants.

[19] Sansone C., Pistelli L., Calabrone L., Del Mondo A., Fontana A., Festa M., Noonan D.M., Albini A., Brunet C. (2023). The Carotenoid Diatoxanthin Modulates Inflammatory and Angiogenesis Pathways In Vitro in Prostate Cancer Cells. Antioxidants.

[20] Goss R., Lepetit B. (2015). Biodiversity of NPQ. J. Plant Physiol..

[21] Lavaud J., Goss R., Demmig-Adams B., Garab G., Adams I.W., Govindjee (2014). The Peculiar Features of Non-Photochemical Fluorescence Quenching in Diatoms and Brown Algae. Non-photochemical Quenching and Energy Dissipation in Plants, Algae and Cyanobacteria.

[22] Sharma A.K., Nymark M., Sparstad T., Bones A.M., Winge P. (2018). Transgene-Free Genome Editing in Marine Algae by Bacterial Conjugation—Comparison with Biolistic CRISPR/Cas9 Transformation. Sci. Rep..

[23] Serif M., Dubois G., Finoux A.L., Teste M.A., Jallet D., Daboussi F. (2018). One-Step Generation of Multiple Gene Knock-Outs in the Diatom *Phaeodactylum Tricornutum* by DNA-Free Genome Editing. Nat. Commun..

[24] Araújo R., Vázquez Calderón F., Sánchez López J., Azevedo I.C., Bruhn A., Fluch S., Garcia Tasende M., Ghaderiardakani F., Ilmjärv T., Laurans M. (2021). Current Status of the Algae Production Industry in Europe: An Emerging Sector of the Blue Bioeconomy. Front. Mar. Sci..

[25] Dautermann O., Lyska D., Andersen-Ranberg J., Becker M., Fröhlich-Nowoisky J., Gartmann H., Krämer L.C., Mayr K., Pieper D., Rij L.M. (2020). An Algal Enzyme Required for Biosynthesis of the Most Abundant Marine Carotenoids. Sci. Adv..

[26] Bai Y., Cao T., Dautermann O., Buschbeck P., Cantrell M.B., Chen Y., Lein C.D., Shi X., Ware M.A., Yang F. (2022). Green Diatom Mutants Reveal an Intricate Biosynthetic Pathway of Fucoxanthin. Proc. Natl. Acad. Sci. USA.

[27] Cao T., Bai Y., Buschbeck P., Tan Q., Cantrell M.B., Chen Y., Jiang Y., Liu R.-Z., Ries N.K., Shi X. (2023). An Unexpected Hydratase Synthesizes the Green Light-Absorbing Pigment Fucoxanthin. Plant Cell.

[28] Bowler C., Allen A.E., Badger J.H., Grimwood J., Jabbari K., Kuo A., Maheswari U., Martens C., Maumus F., Otillar R.P. (2008). The *Phaeodactylum* Genome Reveals the Evolutionary History of Diatom Genomes. Nature.

[29] Coesel S., Obornik M., Varela J., Falciatore A., Bowler C. (2008). Evolutionary Origins and Functions of the Carotenoid Biosynthetic Pathway in Marine Diatoms. PLoS ONE.

[30] Eilers U., Dietzel L., Breitenbach J., Büchel C., Sandmann G. (2016). Identification of Genes Coding for Functional Zeaxanthin Epoxidases in the Diatom *Phaeodactylum Tricornutum*. J. Plant Physiol..

[31] Dautermann O., Lohr M. (2017). A Functional Zeaxanthin Epoxidase from Red Algae Shedding Light on the Evolution of Light-harvesting Carotenoids and the Xanthophyll Cycle in Photosynthetic Eukaryotes. Plant J..

[32] Karas B.J., Diner R.E., Lefebvre S.C., McQuaid J., Phillips A.P.R., Noddings C.M., Brunson J.K., Valas R.E., Deerinck T.J., Jablanovic J. (2015). Designer Diatom Episomes Delivered by Bacterial Conjugation. Nat. Commun..

[33] Dima O., Inzé D. (2021). The Role of Scientists in Policy Making for More Sustainable Agriculture. Curr. Biol..

[34] Voigt B. (2023). EU Regulation of Gene-Edited Plants—A Reform Proposal. Front. Genome Ed..

[35] Jia X., Zhang Q., Jiang M., Huang J., Yu L., Traw M.B., Tian D., Hurst L.D., Yang S. (2021). Mitotic Gene Conversion Can Be as Important as Meiotic Conversion in Driving Genetic Variability in Plants and Other Species without Early Germline Segregation. PLoS Biol..

[36] Nymark M., Finazzi G., Volpe C., Serif M., de Miranda Fonseca D., Sharma A., Sanchez N., Sharma A.K., Ashcroft F., Kissen R. (2023). Loss of CpFTSY Reduces Photosynthetic Performance and Affects Insertion of PsaC of PSI in Diatoms. Plant Cell Physiol..

[37] Nymark M., Volpe C., Hafskjold M.C.G., Kirst H., Serif M., Vadstein O., Bones A.M., Melis A., Winge P. (2019). Loss of ALBINO3b Insertase Results in Truncated Light-Harvesting Antenna in Diatoms. Plant Physiol..

[38] Sharma A.K., Nymark M., Flo S., Sparstad T., Bones A.M., Winge P. (2021). Simultaneous Knockout of Multiple LHCF Genes Using Single SgRNAs and Engineering of a High-Fidelity Cas9 for Precise Genome Editing in Marine Algae. Plant Biotechnol. J..

[39] Niyogi K.K., Bjorkman O., Grossman A.R. (1997). Chlamydomonas Xanthophyll Cycle Mutants Identified by Video Imaging of Chlorophyll Fluorescence Quenching. Plant Cell.

[40] Jin E., Feth B., Melis A. (2003). A Mutant of the Green Alga *Dunaliella Salina* Constitutively Accumulates Zeaxanthin under All Growth Conditions. Biotech Bioeng..

[41] Manfellotto F., Stella G.R., Falciatore A., Brunet C., Ferrante M.I. (2020). Engineering the Unicellular Alga *Phaeodactylum Tricornutum* for Enhancing Carotenoid Production. Antioxidants.

[42] Grouneva I., Jakob T., Wilhelm C., Goss R. (2008). A New Multicomponent NPQ Mechanism in the Diatom *Cyclotella Meneghiniana*. Plant Cell Physiol..

[43] Lavaud J., Materna A.C., Sturm S., Vugrinec S., Kroth P.G. (2012). Silencing of the Violaxanthin De-Epoxidase Gene in the Diatom *Phaeodactylum Tricornutum* Reduces Diatoxanthin Synthesis and Non-Photochemical Quenching. PLoS ONE.

[44] Goss R., Ann Pinto E., Wilhelm C., Richter M. (2006). The Importance of a Highly Active and DeltapH-Regulated Diatoxanthin Epoxidase for the Regulation of the PS II Antenna Function in Diadinoxanthin Cycle Containing Algae. J. Plant Physiol..

[45] Lavaud J., Kroth P.G. (2006). In Diatoms, the Transthylakoid Proton Gradient Regulates the Photoprotective Non-Photochemical Fluorescence Quenching beyond Its Control on the Xanthophyll Cycle. Plant Cell Physiol..

[46] Jumper J., Evans R., Pritzel A., Green T., Figurnov M., Ronneberger O., Tunyasuvunakool K., Bates R., Zidek A., Potapenko A. (2021). Applying and Improving AlphaFold at CASP14. Proteins.

[47] Varadi M., Bertoni D., Magana P., Paramval U., Pidruchna I., Radhakrishnan M., Tsenkov M., Nair S., Mirdita M., Yeo J. (2024). AlphaFold Protein Structure Database in 2024: Providing Structure Coverage for over 214 Million Protein Sequences. Nucleic Acids Res..

[48] Guex N., Peitsch M.C. (1997). SWISS-MODEL and the Swiss-Pdb Viewer: An Environment for Comparative Protein Modeling. Electrophoresis.

[49] Larkin M.A., Blackshields G., Brown N.P., Chenna R., McGettigan P.A., McWilliam H., Valentin F., Wallace I.M., Wilm A., Lopez R. (2007). Clustal W and Clustal X Version 2.0. Bioinformatics.

[50] Tamura K., Stecher G., Kumar S. (2021). MEGA11: Molecular Evolutionary Genetics Analysis Version 11. Mol. Biol. Evol..

[51] Le S.Q., Gascuel O. (2008). An Improved General Amino Acid Replacement Matrix. Mol. Biol. Evol..

[52] Nymark M., Sharma A.K., Sparstad T., Bones A.M., Winge P. (2016). A CRISPR/Cas9 System Adapted for Gene Editing in Marine Algae. Sci. Rep..

[53] Nymark M., Sharma A.K., Hafskjold M.C., Sparstad T., Bones A.M., Winge P. (2017). CRISPR/Cas9 Gene Editing in the Marine Diatom *Phaeodactylum Tricornutum*. Bio-Protocol.

[54] Guillard R.R.L., Smith W.L., Chanley M.H. (1975). Culture of Phytoplankton for Feeding Marine Invertebrates. Culture of Marine Invertebrate Animals: Proceedings—1st Conference on Culture of Marine Invertebrate Animals Greenport.

[55] Volpe C., Vadstein O., Andersen G., Andersen T. (2021). Nanocosm: A Well Plate Photobioreactor for Environmental and Biotechnological Studies. Lab Chip.

[56] Luo H., Al-Dahhan M.H. (2004). Analyzing and Modeling of Photobioreactors by Combining First Principles of Physiology and Hydrodynamics. Biotech Bioeng..

[57] Rodriguez F., Chauton M., Johnsen G., Andresen K., Olsen L.M., Zapata M. (2006). Photoacclimation in Phytoplankton: Implications for Biomass Estimates, Pigment Functionality and Chemotaxonomy. Mar. Biol..

