# Supplementary material for: Zeaxanthin epoxidase 3 Knockout Mutants of the Model Diatom Phaeodactylum tricornutum Enable Commercial Production of the Bioactive Carotenoid Diatoxanthin
Source: Mar Drugs. 2024 Apr 19;22(4):185. doi: 10.3390/md22040185 (PMC11051370; doi:10.3390/md22040185)
Supplement: Supplementary file 1 [file marinedrugs-22-00185-s001.zip › Supplementary Figures_revised.pdf]

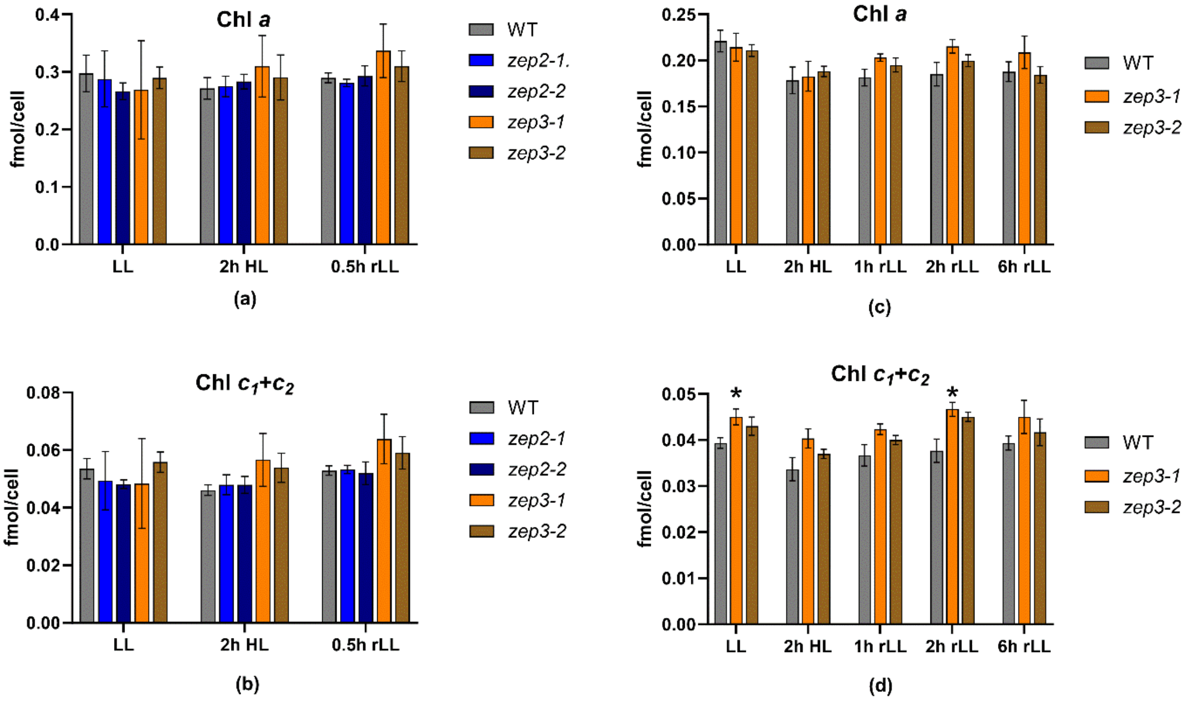

Figure S1. Chlorophyll (Chl) *a* and Chl $c_1 + c_2$ concentration in WT, *zep2* and *zep3* mutant lines. WT, *zep2* and *zep3* cultures were acclimated to low light (LL; 35 µmol photons m^{-2} s^{-1}), exposed to 2 h of high light (HL; 450-500 µmol photons m^{-2} s^{-1}) before being returned to LL for 0.5h (rLL). The resulting (a) Chl *a* and (b) Chl *c1* + *c2* cell concentrations are presented as fmol/cell. An additional experiment was performed with only WT and *zep3* lines where the rLL period was prolonged to 1 h, 2 h and 6 h. Corresponding pigment concentrations are presented in (c) Chl *a* and (d) Chl *c1* + *c2*. All results are presented as means of three biological replicates ± SD. Asterisks describe significant differences between *zep* mutants and WT as indicated by two-way ANOVA with Dunnett's multiple comparison tests ($P < 0.05$).
